# Improving methods to measure comparable mortality by cause (IMMCMC): gold standard verbal autopsy dataset

**DOI:** 10.1186/s13104-021-05834-y

**Published:** 2021-11-23

**Authors:** Riley H. Hazard, Hafizur Rahman Chowdhury, Abraham D. Flaxman, Jonathan C. Joseph, Nurul Alam, Ian Douglas Riley, Peter Kim Streatfield, Hebe Gouda, Seri Maraga, Patricia Rarau, Diozele Sanvictores, Veronica Tallo, Marilla Lucero, Alan D. Lopez

**Affiliations:** 1 School of Population and Global Health, University of Melbourne, Parkville, VIC Australia; 2 Institute for Health Metrics and Evaluation, University of Washington, Seattle, Washington, USA; 3 International Center for Diarrhoeal Disease Research, Dhaka, Bangladesh; 4 School of Public Health, University of Queensland, Brisbane, QLD Australia; 5 PNG Institute of Medical Research, Goroka, Papua New Guinea; 6 Research Institute for Tropical Medicine, Muntinlupa City, Philippines; 7 Department of Health Metrics Science, University of Washington, Seattle, WA USA

**Keywords:** Verbal autopsy, Gold standard, Cause of death

## Abstract

**Objectives:**

Gold standard cause of death data is critically important to improve verbal autopsy (VA) methods in diagnosing cause of death where civil and vital registration systems are inadequate or poor. As part of a three-country research study—Improving Methods to Measure Comparable Mortality by Cause (IMMCMC) study—data were collected on clinicopathological criteria-based gold standard cause of death from hospital record reviews with matched VAs. The purpose of this data note is to make accessible a de-identified format of these gold standard VAs for interested researchers to improve the diagnostic accuracy of VA methods.

**Data description:**

The study was conducted between 2011 and 2014 in the Philippines, Bangladesh, and Papua New Guinea. Gold standard diagnoses of underlying causes of death for deaths occurring in hospital were matched to VAs conducted using a standardized VA questionnaire developed by the Population Health Metrics Consortium. 3512 deaths were collected in total, comprised of 2491 adults (12 years and older), 320 children (28 days to 12 years), and 702 neonates (0–27 days).

## Objective

Knowledge on cause of death are critically important to better inform health policy but often these data are lacking or missing in developing countries. Verbal autopsy (VA) is a practical method that involves administering a structed questionnaire to the family of the deceased to reveal information about the signs and symptoms surrounding deaths occurring outside hospitals. The results of these interviews were traditionally reviewed by physicians but are increasingly being interpreted by computer algorithms to predict cause of death.

One approach to developing VA algorithms relies on VAs that have an associated “gold standard” cause of death assigned using a rigorous criteria by a group of physicians. The performance of VA algorithms can also be tested using these gold standard datasets. The gold standard cause of death must be determined using the highest amount of clinical evidence and agreed upon by multiple physicians. Because gold standard cause of death requires a high level of clinical evidence, these deaths must occur in hospitals with access to laboratory and imaging investigations. The VAs are then conducted for these deaths.

The Population Health Metrics Research Consortium (PHMRC) gold standard VA dataset has been used to develop and test the performance of many VA algorithms [1]. It collected over 12,000 gold standard VAs with 7836 adults, 2075 children, 1629 neonates, and 1002 stillbirths in the Philippines, Mexico, Tanzania and India. In this data note, we highlight the release of a second gold standard VA dataset: Improving Methods to Measure Comparable Mortality by Cause (IMMCMC) dataset. This dataset was used in a recent publication to assess how the addition of this dataset to the PHMRC dataset influenced the algorithm associations and performance [2]. The IMMCMC dataset was developed using a similar protocol as the PHMRC dataset and can be used to further the development and performance of other VA algorithms.

## Data description

### Gold standard cause of death

The IMMCMC study gathered gold standard VAs between 2011 and 2014 using the PHMRC long form verbal autopsy instrument [1]. For deaths that occurred in-hospital, a medical record review was conducted to establish, as confidently as possible, the true cause of death by applying the PHMRC “gold standard” diagnostic criteria. The same cause of death list as the PHMRC study was also used.

To evaluate the quality of gold standard diagnoses, clinical categories were established according to the degree to which the information from the medical record provided sufficient certainty to determine whether the death could be used as part of the VA validation study. GS1, GS2A, and GS2B provided the highest diagnostic certainty based on laboratory tests, clinical imaging, and documented illness signs (Additional files 1 and 2) (Table 1). In Papua New Guinea and the Philippines, medical records were reviewed by two unblinded physicians and disagreements were settled by a study physician using the medical data and audit form (Additional file 3). In Bangladesh, a single physician reviewed medical records and allocated an underlying cause of death using the medical data and audit form. There was no step for settling disagreements in Bangladesh.

**Table 1:** Overview of data files/data sets

Label	Name of data file/data set	File types (file extension)	Data repository and identifier (DOI or accession number)
Data file 1	Gold Standard Verbal Autopsy Dataset 2011–2014	.csv	Zenodo https://doi.org/10.5281/zenodo.5573635 [5]
Data file 2	Codebook	.csv	Zenodo https://doi.org/10.5281/zenodo.5573635 [5]
Additional file 1	Gold standard definitions in the IMMCMC study for adults	.docx	Zenodo https://doi.org/10.5281/zenodo.5573635 [5]
Additional file 2	Gold standard definitions in the IMMCMC study for children and neonates	.docx	Zenodo [5] https://doi.org/10.5281/zenodo.5573635 [5]
Additional file 3	Medical Data and Audit Form	.docx	Zenodo https://doi.org/10.5281/zenodo.5573635 [5]

In this data note, we present 3512 cases that met GS1, GS2A, and GS2B criteria. This dataset included 2491 adults (12 years and older), 320 children (28 days to 12 years), and 702 neonates (0–27 days). The majority of the cases were from Bohol, Philippines, which contributed 2384 VAs, 1070 VAs were from Bangladesh, and 58 were from Papua New Guinea.

### Data collection sites

Bohol, Philippines is an island province with a population of about 1.2 million. It has 47 municipalities and one city (Tagbilaran City). VAs were collected for all deaths in 11 of the municipalities which had been selected as clusters with probability of selection proportional to size. Deaths were identified by the capture-recapture method from three sources: the civil register, health center records, and the Catholic Church parish registers [2, 3]. Study nurses and trained support personnel conducted the VA interviews.

The Matlab Subdistrict in Chandpur District of Bangladesh include Matlab Health and Demographic Sousveillance (HDSS) which has a total population of approximately 225,000. VAs were collected from all deaths in the Matlab HDSS during the study period [2, 3]. A team of trained field research supervisors (non-medical) conducted the VA interviews.

Deaths in Papua New Guinea were identified from the Partnerships in Health HDSS in four sites: Hiri, Central Province; Hides, Southern Highlands Province; Asaro Valley, Eastern Highlands Province; Karkar, Madang Province [2, 3]. A team of research nurses and health extension officers conducted the VA interviews.

### Verbal autopsies

VAs were collected using the PHMRC VA instrument [1]. VAs were conducted by trained support staff. Training consisted of a 1-week period followed by supervision. VAs were collected a maximum of 12 months after death in order to minimize recall bias.

### Data processing

Data file 1 contains the responses using the full PHMRC verbal autopsy instrument in addition to the gold standard diagnosis using both the full (46) and reduced (34) cause list for adults. Variables also include the country of death, age group, and gold standard category. For data privacy purposes, all patient identifiers, names, languages, birth dates, and death dates were removed. Ages over 80 were truncated. The transcribed open narrative response and all questions that involved a free text response were replaced with only the key words that were identified in PHMRC dataset. The PHMRC VA responses are also included in the dataset for comparison and denoted using as “PHMRC” under the “study” variable while the IMMCMC responses are denoted under “NHMRC”.

## Limitations

The IMMCMC dataset is one of the largest gold standard VA datasets to be released. The data collection process followed the same protocol as the PHMRC gold standard VA dataset which has been widely used in developing and validating VA algorithms. The IMMCMC dataset has some of the same limitations at the PHMRC dataset in that it only examined hospitals deaths, as opposed to the community deaths that VA intends to measure. Additionally, the IMMCMC dataset was limited to specific causes set out in the PHMRC study. Finally, since the IMMCMC dataset used the PHMRC VA instrument, it does not contain some of the questions from the WHO VA questionnaire, which may be necessary for other VA algorithms [4].

## Data Availability

The data described in this Data Note can be freely and openly accessed at https://doi.org/10.5281/zenodo.5573635 [5]. Please see Table 1 for details and links to the dataset.

## Abbreviations

VA: Verbal autopsy
PHMRC: Population Health Metrics Consortium
IMMCMC: Improving methods to measure comparable mortality by cause

## References

[1] Murray CJ, Lopez AD, Black R, Ahuja R, Ali SM, Baqui A (2011). Population Health Metrics Research Consortium gold standard verbal autopsy validation study: design, implementation, and development of analysis datasets. Popul Health Metr..

[2] Chowdhury HR, Flaxman AD, Joseph JC, Hazard RH, Alam N, Riley ID (2019). Robustness of the Tariff method for diagnosing verbal autopsies: impact of additional site data on the relationship between symptom and cause. BMC Med Res Methodol..

[3] Williams GM, Riley ID, Hazard RH, Chowhury HR, Alam N, Streafield PK (2019). On the estimation of population cause-specific mortality fractions from in-hospital deaths. BMC Med..

[4] Nichols EK, Byass P, Chandramohan D, Clark SJ, Flaxman AD, Jakob R (2018). The WHO 2016 verbal autopsy instrument: An international standard suitable for automated analysis by InterVA, InSilicoVA, and Tariff 2.0. PLOS Med..

[5] Hazard RH, Chowdhury HR, Flaxman AD, Alam N, Riley ID, Streatfield PK, et al. Improving Methods to Measure Comparable Mortality by Cause—Gold Standard Verbal Autopsy Data 2011–2014. Zenodo. 2020.

