# Correction to: Improving methods to measure comparable mortality by cause (IMMCMC): gold standard verbal autopsy dataset

**DOI:** 10.1186/s13104-022-05909-4

**Published:** 2022-01-24

**Authors:** Riley H. Hazard, Hafzur Rahman Chowdhury, Abraham D. Flaxman, Jonathan C. Joseph, Nurul Alam, Ian Douglas Riley, Peter Kim Streatfeld, Hebe Gouda, Seri Maraga, Patricia Rarau, Diozele Sanvictores, Veronica Tallo, Marilla Lucero, Alan D. Lopez

**Affiliations:** 1 School of Population and Global Health, University of Melbourne, Parkville, VIC Australia; 2 Institute for Health Metrics and Evaluation, University of Washington, Seattle, WA USA; 3 International Center for Diarrhoeal Disease Research, Dhaka, Bangladesh; 4 School of Public Health, University of Queensland, Brisbane, QLD Australia; 5 PNG Institute of Medical Research, Goroka, Papua New Guinea; 6 Research Institute for Tropical Medicine, Muntinlupa City, Philippines; 7 Department of Health Metrics Science, University of Washington, Seattle, WA USA

## Correction to: BMC Research Notes (2021) 14:422 https://doi.org/10.1186/s13104-021-05834-y

Following the publication of the original article [1], the authors requested to amend the article title from “Improving methods to measure comparable mortality cause (IMMCMC) gold standard verbal autopsy dataset” to “Improving methods to measure comparable mortality by cause (IMMCMC): gold standard verbal autopsy dataset”.

The correct title is included in this Correction and has already been updated in the original article.
